# DIS3L2 Gene Mutation Causes the Perlman Syndrome of Overgrowth and Wilms Tumor Susceptibility

**DOI:** 10.7759/cureus.49777

**Published:** 2023-12-01

**Authors:** Hussain A Al Ghadeer, Fouad A Alghazal, Marwah A Alessa, Jinan A Alghafli, Ghufran I Alkhalaf, Hassan N Bumejdad, Rabab M Alherz, Razan A Alshaikh Saleh, Khulud A Almumtin, Ahmed K Abu Sinah

**Affiliations:** 1 Paediatrics, Maternity and Children Hospital, Al-Ahsa, SAU; 2 Neonatology, Maternity and Children Hospital, Al-Ahsa, SAU; 3 Pediatrics, Maternity and Children Hospital, Al-Ahsa, SAU; 4 Pediatrics, Maternity and Children Hospital, Al-Hofuf, SAU; 5 Medicine and Surgery, King Faisal University, Al-Ahsa, SAU; 6 Pediatrics, Almaarefa University, Riyadh, SAU; 7 Pediatrics, King Faisal University, Al-Ahsa, SAU

**Keywords:** polyhydramnios, saudi arabia, nephroblastomatosis, overgrowth, dis3l2 gene, perlman syndrome

## Abstract

The deletion of the DIS3L2 gene causes the extremely uncommon congenital overgrowth syndrome, known as Perlman syndrome, which is autosomal recessive. Polyhydramnios, macrosomia, facial dysmorphism, renal dysplasia, and several congenital abnormalities with Wilms tumor propensity are its defining features. Beckwith-Wiedemann syndrome (BWS), prune belly syndrome (PBS), and Simpson-Golabi-Behmel syndrome (SGBS1) have certain similar clinical characteristics with Perlman syndrome. The syndrome is often associated with a high neonatal mortality rate and there are few reports of long-term survivors. Here, we present a case with the classic clinical features of Perlman syndrome and a DIS3L2 gene deletion that was discovered prenatally.

## Introduction

In the 1970s and 1980s, Perlman syndrome was first reported. Polyhydramnios, fetal overgrowth, facial dysmorphism with a round face, wide forehead, deep-set eyes, broad nasal bridge, hypertelorism, everted upper lip, high arched palate, low-set ears, and visceromegaly are all characteristics of Perlman syndrome [1-3]. Neonatal mortality is high due to renal abnormalities, and the majority of infants who survive the neonatal period will develop Wilms tumor. For families at risk of Perlman syndrome, prenatal diagnosis is feasible. The initial indicators of Perlman syndrome may include fetal overgrowth, specifically an occipitofrontal head circumference larger than the 90th centile for gestational age, which is related to polyhydramnios. Prompt recognition and appropriate intervention are required to prevent the high morbidity and mortality in Perlman syndrome [4].

## Case presentation

This report presents the case of a six-month-old Saudi female, delivered by cesarean section at 37 weeks of gestation due to polyhydramnios and fetal distress. The patient's mother was 24 years old, healthy, gravida 2 para 1 on regular follow-up with perinatology due to the history of infant death at the age of seven months due to multiple congenital anomalies in the form of congenital heart disease (cardiomegaly, aneurysm of the atrial septum, isomerism) and bilateral hydronephrosis with no final diagnosed reached. During this pregnancy, at the gestational age of 32 weeks, the ultrasound scan showed bilateral hydronephrosis (right kidney 2.7 cm, left kidney 1.8 cm). The fetal biometry, anatomical scan, placenta, and liquor were within normal limits.

The mother had no history of exposure to teratogenic substances, medications, and radiation during this pregnancy. The parents are consanguine (1st degree), and there was no family history of genetic, metabolic, or inherited diseases. At the gestational age of 36 weeks, amniocentesis confirmed the genetic mutation in DIS3L2 via single nucleotide polymorphisms (SNP) of the fetus. 

**Figure 1:**
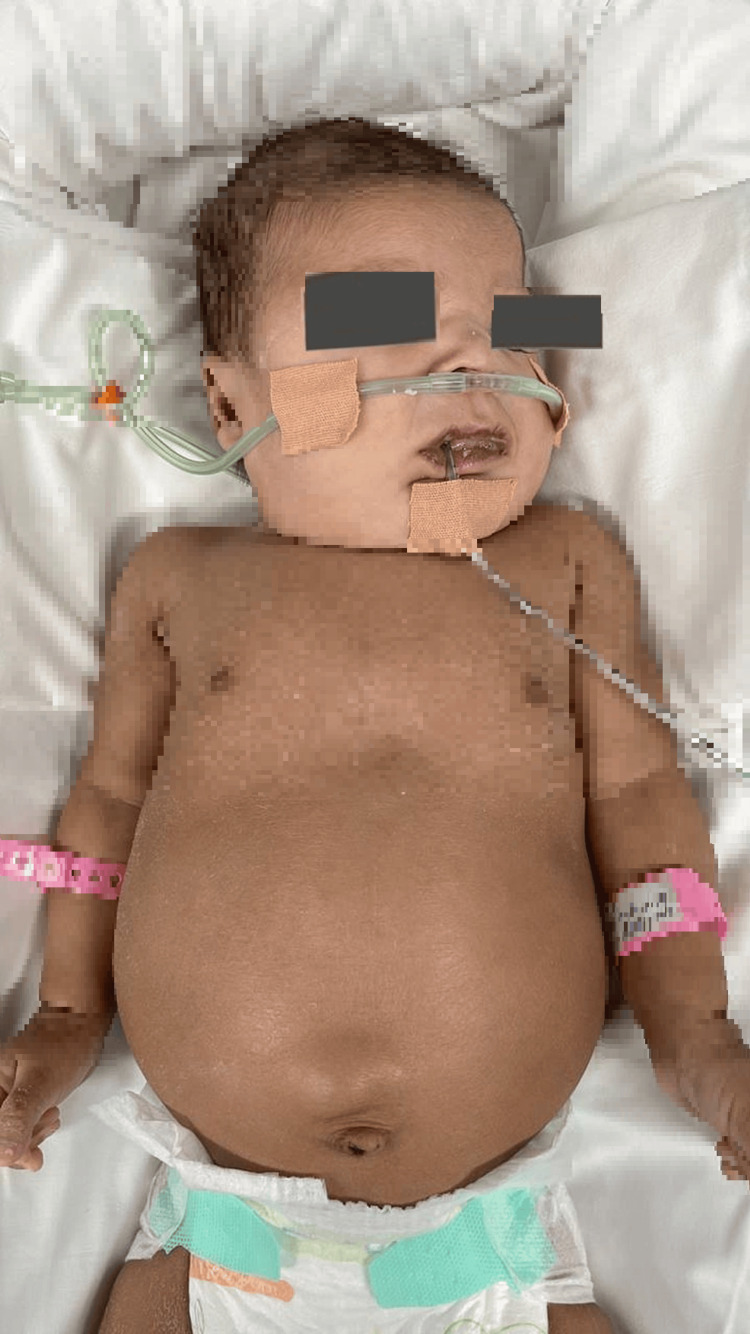
The clinical features of the patient Prominent forehead, rounded face, depressed nasal bridge, V-shaped upper lip, highly arched palate, and distended abdomen.

At the time of birth, The Apgar score was 8 at both 1 minute and 5 minutes. The anthropometric measurement revealed weight was 4 kg (>95th centile), height 52 cm (75th centile), head circumference 34 cm (25th centile). On neonatal evaluation, the newborn showed dysmorphism: prominent forehead, rounded face, deep-set eyes, hypertelorism, epicanthic folds, depressed nasal bridge, V-shaped upper lip, and highly arched palate. The abdominal examination revealed enlarged and palpable kidneys with paraumbilical hernia, and no hepatosplenomegaly (Figure 1). A 3/6 systolic murmur in the left sternal border and axial hypotonia were also found. The infant was initially kept on a nasal cannula then deteriorated over the hospital course till intubated and ventilated due to frequent attacks of apnea. She required glucose infusions for a few days due to hypoglycemia and nasogastric tube feeding due to poor sucking.

**Figure 2:**
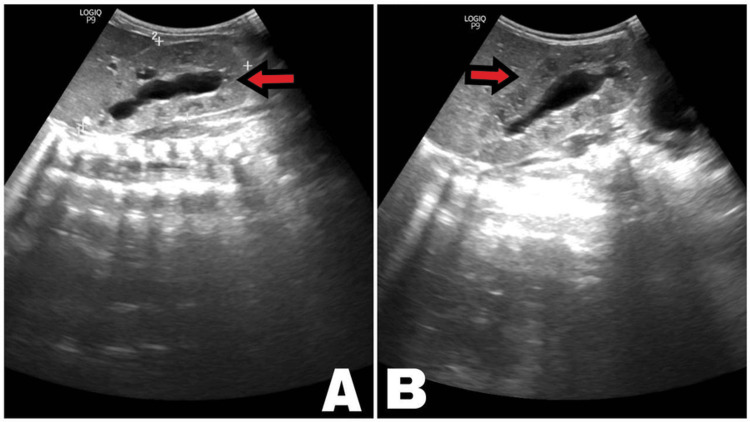
Renal ultrasound A: right kidney, B: left kidney The image shows enlarged kidneys for age (right 7.6 cm x 4.4 cm, left 8.4 cm x 4.1 cm) with a mild increase in cortical echogenicity and hydronephrosis (right 1.7 cm, left 1.8 cm).

**Figure 3:**
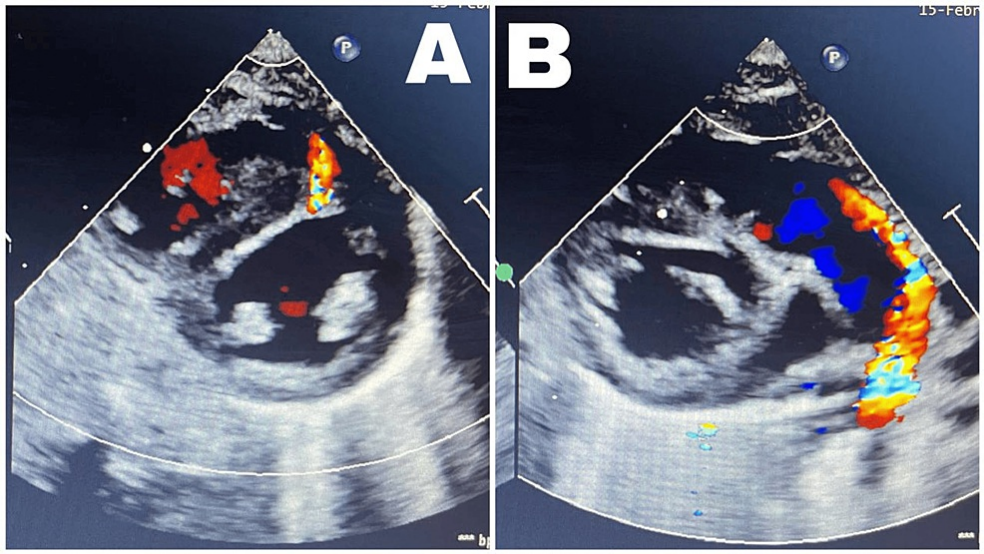
Echocardiography An echocardiography study revealed a small anterior muscular ventricular septal defect (VSD) (A) and moderate patent ductus arteriosus (PDA) (B).

Abdominal ultrasonography showed enlarged kidneys for age (right 7.6 cm x 4.4 cm, left 8.4 cm x 4.1 cm) with a mild increase in cortical echogenicity and hydronephrosis (right 1.7 cm, left 1.8 cm), with no other organomegaly (Figures 2A-2B). No abnormalities were detected by Micturating Cystourethrogram (MCUG), brain ultrasound, and magnetic resonance imaging (MRI). Small anterior muscular ventricular septal defect (VSD) and moderate patent ductus arteriosus (PDA), were found via Echocardiography (Figures 3A-3B).

The infant was admitted to the neonatal intensive care unit for four months and then was discharged on an oxygen 2 L nasal cannula, maintaining the saturation on room air. The patient had recurrent readmission four times after discharge due to respiratory distress, attacks of apnea, poor feeding, and frequent aspiration pneumonia. Developmentally, the infant had generalized hypotonia, an absence of social smiles, or social interaction.

**Figure 4:**
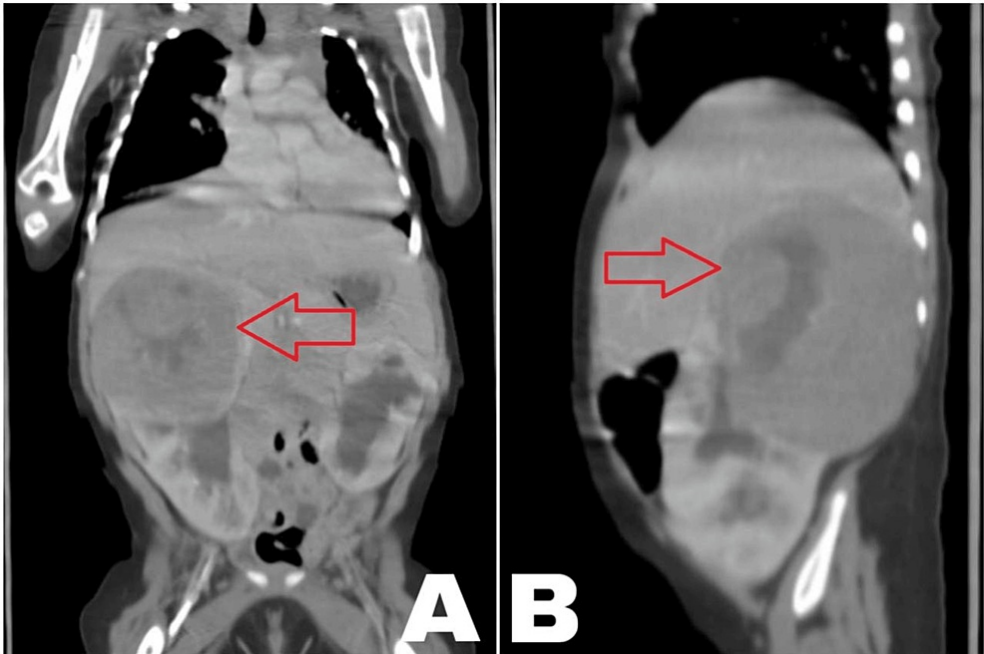
CT scan of the abdomen Coronal view (A): shows a large, poorly enhancing mass with central necrosis arising from the upper pole of the right kidney. Please note the bilateral moderate hydronephrosis. Sagittal view (B): The upper pole of the right kidney is largely replaced by the mass with a bulging contour causing a mass effect upon the right hepatic lobe.

During the follow-up at the age of six months, an abdominal mass suggestive of Wilms tumor was discovered (Figures 4A-4B). The patient was transferred to a tertiary care center for further management.

## Discussion

Overgrowth syndromes are a broad class of disorders characterized by uncontrollable growth. During pregnancy, ultrasound scans can detect overgrowth throughout the fetus, resulting in excessive length and/or weight at delivery (defined as >90th percentile or 2 standard deviations above the mean weight or length for gestational age at birth). Growth management is a complex process that involves a number of variables, including those with genetic or epigenetic, endocrine, and metabolic origins [5,6]. Perlman and colleagues first discovered the Perlman syndrome in five of six siblings who were born into a consanguineous, two-generation Jewish-Yemenite family [2]. In this report, we discussed a female girl diagnosed with Perlman syndrome perinatally through amniocentesis.

Fetal macrosomia, visceromegaly, hypotonia, hypertrophy of the islets of Langerhans, cryptorchidism, and facial dysmorphism, as evidenced by a wide forehead, a broad depressed nasal bridge, a long upper lip with an inverted V-shape, and micrognathia, are features that characterize Perlman syndrome. Two other signs of Perlman syndrome include mental impairment and corpus callosum agenesis. Several isolated abnormalities exist, including a diaphragmatic hernia, an interrupted aortic arch, an aberrant left coronary artery, polysplenia, volvulus, and distal ileal atresia [7,8]. Nephromegaly, hydronephrosis, renal dysplasia, hamartomas, nephroblastomatosis, and Wilms tumor growth are among the renal complications associated with Perlman syndrome. Prenatal ultrasonography screening might reveal enlarged hyperechogenic kidneys. In our patient, who had normal renal functioning on the first days of birth, nephromegaly was diagnosed. According to Fahmy et al. [8] the age of diagnosis of Wilms tumor was four days, eight months, 10 months, and four years. As a result, babies with Perlman syndrome must be monitored with routine abdominal sonography every three months until the age of five years to detect a Wilms tumor early. Piccione et al. [9] reported a Wilms tumor identified at one year and eight months and effectively treated with chemotherapy, with no recurrence or metastases. In contrast, Neri et al. [1] reported a case with a recurrence of the Wilms tumor at the original after one year following surgical resection. In Addison, a pulmonary metastasis was found at the age of six years and six months of that patient.

The clinical and radiological follow-up for our patient at the age of six months was suggestive of a Wilms tumor. Clinical similarities between Perlman syndrome and two other newborn overgrowth syndromes, Beckwith-Wiedemann Syndrome (BWS) and Simpson-Golabi-Behmel Syndrome (SGBS) have been recognized. Perlman syndrome can be misdiagnosed as BWS due to its similarity until the histological finding of renal biopsy or appearance in the sibship [10,11]. The current report has none of the well-known BWS features, including macroglossia, an aberrant tongue, creases in the ears, or hemi hyperplasia. Being a female and not having polydactyly or a cleft lip and palate lessen the likelihood of SGBS.

In the second and third trimesters, the most common antenatal sonographic abnormalities include polyhydramnios, macrosomia, nephromegaly, and visceromegaly. Fetal ascites is another prenatal abnormality that has been reported on occasion [10,12]. The prevalence is one in one million. Approximately 30 patients have been described in the literature [13]. Perlman syndrome (Online Mendelian Inheritance in Man (OMIM); 267000) is an autosomal recessive disorder caused by mutations in the DIS3L2 gene. The DIS3L2 gene product possesses ribonuclease activity and is related to the DIS3 RNA exosome component. Although the particular targets of DIS3L2 have yet to be identified, DIS3L2 knockdown is related to defects of cell growth and division in cellular models [3]. In our case, the prenatal confirmation of Perlman syndrome was verified by the microarray discovery of a deletion of the DIS3L2 gene on chromosome 2q37.1.

Perlman syndrome carries a poor prognosis with a high mortality rate during infancy. Sepsis or respiratory insufficiency is the leading cause of death. There have been few reports of long-term survivors. Piccione et al. [9] reported an exceptional case of a nine-year-old girl who had normal neurological and cognitive development. The patient in this case was oxygen-dependent when she was discharged at the age of four months, but he has required re-admission due to aspiration and frequent episodes of apnea and at the age of six months was discovered to have a Wilms tumor. The family was counseled regarding the anticipated results and the suggestion that in vitro fertilization be used for the next pregnancy.

## Conclusions

Several variables involved in cell proliferation and/or the control of gene expression are predominantly disrupted on an epigenetic and genetic level in overgrowth disorders. The increased risk of tumors in overgrowth syndromes may be explained by the frequent observation of anomalies in the same genes and/or pathways that produce overgrowth syndromes in tumors. Due to variations in tumor surveillance, the type of molecular test, and genetic counseling, it is important to differentiate between these disorders in order to provide the best patient care.
